# Neurologic Features with Pathogenic Copy Number Variants

**DOI:** 10.15844/pedneurbriefs-34-20

**Published:** 2020-12-18

**Authors:** Jason Coryell

**Affiliations:** 1 Departments of Pediatrics and Neurology, Oregon Health & Sciences University, Portland, OR

**Keywords:** Comparative Genomic Hybridization, Genetic, Pediatric Neurology

## Abstract

Investigators from Children’s Hospital at Westmead, University of Sydney, performed a retrospective review (2006-2012) of the diagnostic yield of array comparative genomic hybridization (aCGH) among 555 children with diverse neurologic phenotypes in whom a genetic etiology was suspected [1].

Investigators from Children’s Hospital at Westmead, University of Sydney, performed a retrospective review (2006-2012) of the diagnostic yield of array comparative genomic hybridization (aCGH) among 555 children with diverse neurologic phenotypes in whom a genetic etiology was suspected [1]. Pathogenicity of copy number variants (CNV) was classified according to previously published guidelines [2]. Forty-seven patients (8.6%) had pathogenic variants. The neurologic phenotype was divided into 17 broad categories. Those with significantly increased odds ratios of a pathogenic CNV included: global developmental delay (DD) [OR 3.69], dysmorphism [OR 2.75], cortical visual impairment [2.73], and microcephaly [OR 2.16]. Logistic regression analysis showed an additive effect of multiple phenotypic categories being more likely associated with a pathogenic CNV (OR 1.18). The combination of developmental delay/intellectual disability with dysmorphism and abnormal head circumference showed the greatest effect among combined categories (OR 2.86). Epilepsy, cerebral palsy, tone abnormality, ataxia, movement disorder, psychiatric comorbidity, and abnormal neuro-diagnostics (MRI brain or spine, EEG) were not independently predictive for pathogenic CNV. [1]

COMMENTARY. This study is in line with multiple prior studies showing increased frequency (~15%) of pathogenic CNVs in individuals with developmental delay (DD)/ intellectual disability (ID) [3]. Pathogenic CNVs have also been shown at higher rates in those with multiple congenital anomalies (17%) [4]. Additionally, >50% of individuals with pathogenic CNVs may have dysmorphic features when refined phenotyping is applied [5].

The authors suggest that the diagnostic yield of aCGH warrants this as a first-tier test in pediatric neurology patients; however, aCGH is perhaps best suited for a targeted population: including those with DD/ID, dysmorphic features, multiple congenital anomalies, or microcephaly. Other studies addressing specific neuro-phenotypes, such as epilepsy or weakness, show a higher diagnostic yield with whole-exome sequencing (WES) or targeted panels. For example, in pediatric epilepsy patients, a meta-analysis revealed a diagnostic yield of 45% for WES, 23% for a targeted panel (TP), and 8% for CGH. A cost-effectiveness analysis indicated that a tiered testing system was cheaper when the initial test was WES or TP, rather than aCGH [6]. Similarly, the diagnostic yield of WES within a pediatric neuromuscular clinic was 39% [7].

This chart review predates the increased use of next-generation sequencing panels or WES. As the authors indicate, the increasing use of WES as a first test will identify many CNVs previously detected on aCGH. If there is a high a priori suspicion that the phenotype is more consistent with a CNV than a single gene disorder, aCGH could be a more rapid and cost-effective approach for that subset of neurology patients.

This article contributes to pediatric neurogenetics literature by helping to narrow the spectrum of neuro-phenotypes for whom CGH may be the best initial test.

## Disclosures

The author has declared that no competing interests exist.
